# HACE1 builds molecular crosstalks between rare diseases and (more) common disorders

**DOI:** 10.1002/ctm2.922

**Published:** 2022-06-09

**Authors:** Frederic Tort

**Affiliations:** ^1^ Secció d'Errors Congènits del Metabolisme‐IBC, Servei de Bioquímica i Genètica Molecular Hospital Clínic de Barcelona, IDIBAPS, CIBERER Barcelona Spain

**Keywords:** HACE1, rare disorders, mitochondria, cancer

1

*HACE1* encodes for an HECT domain and ankyrin repeat‐containing ubiquitin ligase, which is involved in tagging specific proteins for subcellular localization or degradation.  A recent study reported the implication of HACE1 in cisplatin‐induced cell death in gastric cancer by regulating cyclin C localization.^1^ This study provides solid data demonstrating that HACE1 specifically ubiquitylates Cyclin C at Lys11, which promotes the re‐localization of this protein on the mitochondrial surface. However, the association of HACE1 with cancer is not new, since promoter hypermethylation of *HACE1* gene and reduced protein expression have been observed in several tumour types. In fact, HACE1 is considered to act as a tumour suppressor whose downregulation could enhance tumour growth and progression, whereas its overexpression could inhibit tumour development.^2^ However, the implication of HACE1 in human disease is not limited to cancer. This commentary extends the discussion about the molecular function of HACE1 beyond its role in tumour biology and highlights the importance of establishing crosstalks between rare diseases and more common disorders.

*HACE1* germline mutations are associated with a rare genetic disorder with autosomal recessive inheritance pattern, classified in the Online Mendelian Inheritance in Man (OMIM) database as spastic paraplegia and psychomotor retardation with or without seizures (SPPRS) (MIM #616756). This disease was discovered in 2015 in the context of a comprehensive genetic study involving 4295 patients with undiagnosed developmental diseases.^3^ In this study, the authors identified six individuals, from four unrelated families, carrying *HACE1* loss of function mutations and postulate, for the first time, that HACE1 deficiency could be associated with an inherited genetic condition. In the following years, other studies identified additional HACE1 patients and at present, according to a recent bibliographic search in Pubmed, the number of reported individuals has increased up to 20.^4^, ^5^, ^6^, ^7^, ^8^ The affected individuals presented in infancy or in the first months of life with a variety of symptoms that included intellectual disability, psychomotor retardation, hypotonia, dystonia, spasticity, epilepsy, and ataxia. Brain imaging revealed variable hypoplasia of the corpus callosum, brain atrophy, delayed myelinization, and white matter abnormalities. The molecular mechanisms underlying HACE1 deficiency have not been completely elucidated, but several studies performed in cellular and animal models provided some insights pointing to altered regulation of autophagy and oxidative stress response pathways.^9^, ^10^ On the other hand, a recent study performed in SPPRS primary fibroblasts reported significant abnormalities affecting mitochondrial morphology and network branching. In addition, SPPRS cells showed a strong reduction of the mitophagic flux and a marked accumulation of both the overall and the intramitochondrial oxidative damage due to defective activation of Nrf2‐dependent pathways.^7^ The mitochondrial defects observed in SPPRS patients together with the fact that HACE1 is required for cyclin C mitochondrial translocation reinforces the role of HACE1 in the regulation of mitochondrial physiology. In that sense, the upcoming discovery of novel HACE1 mitochondrial targets will open an exciting scenario that will improve the knowledge about the physiopathology of SPPRS. Reciprocally, the precise delineation of the molecular mechanisms underlying this rare inherited genetic condition will be fundamental to understand cellular processes associated with more common disorders, such as cancer. In this case, many questions will need to be addressed in the near future, such as the potential roles of HACE1‐Nrf2 axis and the (im)balance between autophagy/mitophagy and cell death.

## CONFLICT OF INTEREST

The author declares no conflict of interest.

## References

[1] Jiang HY , Chen YL , Xu XX , et al. Ubiquitylation of cyclin C by HACE1 regulates cisplatin‐associated sensitivity in gastric cancer. Clin Transl Med. 2022;12(3):e770. 35343092 10.1002/ctm2.770 PMC8958351

[2] Zhang L , Anglesio MS , O'Sullivan M , et al. The E3 ligase HACE1 is a critical chromosome 6q21 tumor suppressor involved in multiple cancers. Nat Med. 2007:13:1060‐1069. 17694067 10.1038/nm1621

[3] Akawi N , McRae J , Ansari M , et al. Discovery of four recessive developmental disorders using probabilistic genotype and phenotype matching among 4125 families. Nat Genet. 2015;47:1363‐1369. 26437029 10.1038/ng.3410 PMC5988033

[4] Hollstein R , Parry, DA , Nalbach, L , et al. HACE1 deficiency causes an autosomal recessive neurodevelopmental syndrome. J Med Genet. 2015;52:797‐803. 26424145 10.1136/jmedgenet-2015-103344 PMC4717446

[5] Hariharan N , Ravi S , Pradeep BE , et al. A novel loss‐of‐function mutation in HACE1 is linked to a genetic disorder in a patient from India. Hum Genome Var. 2018;5:1‐4. 29423242 10.1038/hgv.2017.61 PMC5803204

[6] Nagy V , Hollstein R , Pai TP , et al. HACE1 deficiency leads to structural and functional neurodevelopmental defects. Neurol Genet. 2019;5:e330. 31321300 10.1212/NXG.0000000000000330 PMC6561753

[7] Ugarteburu O , Sánchez‐Vilés M , Ramos J , et al. Physiopathological bases of the disease caused by HACE1 mutations: alterations in autophagy, mitophagy and oxidative stress response. J Clin Med. 2020;9:913. 10.3390/jcm9040913 PMC7231286 32225089

[8] Sager G , Turkyilmaz A , Ates EA , et al. HACE1, GLRX5, and ELP2 gene variant cause spastic paraplegies. Acta Neurol Belg. 2022;122(2):391‐399. 33813722 10.1007/s13760-021-01649-7

[9] Rotblat B , Southwell AL , Ehrnhoefer DE , et al. HACE1 reduces oxidative stress and mutant Huntingtin toxicity by promoting the NRF2 response. Proc Natl Acad Sci USA. 2014;111:3032‐3037. 24516159 10.1073/pnas.1314421111 PMC3939919

[10] Zhang L , Chen X , Sharma P , Moon M , et al. HACE1‐dependent protein degradation provides cardiac protection in response to haemodynamic stress. Nat Commun 2014;5:1‐14. 10.1038/ncomms4430 PMC3959209 24614889

